# Developmental Hippocampal Neuroplasticity in a Model of Nicotine Replacement Therapy during Pregnancy and Breastfeeding

**DOI:** 10.1371/journal.pone.0037219

**Published:** 2012-05-15

**Authors:** Ian Mahar, Rosemary C. Bagot, Maria Antonietta Davoli, Sharon Miksys, Rachel F. Tyndale, Claire-Dominique Walker, Marissa Maheu, Sheng-Hai Huang, Tak Pan Wong, Naguib Mechawar

**Affiliations:** 1 Departments of Psychiatry, Neurology and Neurosurgery, Douglas Mental Health University Institute, McGill University, Verdun, Québec, Canada; 2 Departments of Pharmacology and Toxicology and Psychiatry, Centre for Addiction and Mental Health and University of Toronto, Medical Sciences Building, Toronto, Ontario, Canada; 3 Department of Microbiology, College of Basic Medicine, Anhui Medical University, Hefei, Anhui, People's Republic of China; 4 Department of Pharmacology & Therapeutics, Douglas Mental Health University Institute, McGill University, Verdun, Québec, Canada; Radboud University, The Netherlands

## Abstract

**Rationale:**

The influence of developmental nicotine exposure on the brain represents an important health topic in light of the popularity of nicotine replacement therapy (NRT) as a smoking cessation method during pregnancy.

**Objectives:**

In this study, we used a model of NRT during pregnancy and breastfeeding to explore the consequences of chronic developmental nicotine exposure on cerebral neuroplasticity in the offspring. We focused on two dynamic lifelong phenomena in the dentate gyrus (DG) of the hippocampus that are highly sensitive to the environment: granule cell neurogenesis and long-term potentiation (LTP).

**Methods:**

Pregnant rats were implanted with osmotic mini-pumps delivering either nicotine or saline solutions. Plasma nicotine and metabolite levels were measured in dams and offspring. Corticosterone levels, DG neurogenesis (cell proliferation, survival and differentiation) and glutamatergic electrophysiological activity were measured in pups.

**Results:**

Juvenile (P15) and adolescent (P41) offspring exposed to nicotine throughout prenatal and postnatal development displayed no significant alteration in DG neurogenesis compared to control offspring. However, NRT-like nicotine exposure significantly increased LTP in the DG of juvenile offspring as measured *in vitro* from hippocampal slices, suggesting that the mechanisms underlying nicotine-induced LTP enhancement previously described in adult rats are already functional in pups.

**Conclusions:**

These results indicate that synaptic plasticity is disrupted in offspring breastfed by dams passively exposed to nicotine in an NRT-like fashion.

## Introduction

It is estimated that 10–25% of women smoke tobacco during pregnancy [1], [2]. This addiction has well-documented adverse effects on pregnancies, as it is associated with increased occurrences of placenta previa, premature delivery and stillbirth, and is the main cause of low birth weight in Western societies [3]. Furthermore, children born from mothers who smoke are more susceptible to sudden infant death syndrome (SIDS) [4]. They have also been reported to display cognitive deficits [5]–[7], and to run a greater risk of developing psychiatric conditions including anxiety disorders [8] and attention deficit hyperactivity disorder (ADHD) [9], [10]. These studies highlight the vulnerability of developing brains to tobacco smoke that, in addition to the addictive compound nicotine, contains several other neuroactive molecules [11].

In response to the highly publicized risks associated with smoking, combined with the difficulty of quitting, women commonly use smoking cessation aids during pregnancy and breastfeeding [12]. Non-prescription nicotine replacement therapy (NRT) is the most popular of these methods [13], [14], as it can help smokers reduce withdrawal from smoking. Nicotine alone is expected to be less harmful to the fetus than cigarette smoke [15]. Accordingly, recent studies in a Danish cohort found no evidence that NRT increases stillbirth [16] or that it affects offspring birthweight [17]. However, longitudinal data on NRT safety during pregnancy and breastfeeding, and its possible influence on cognition and behavior in offspring, have yet to be produced.

Although hundreds of studies, using a variety of nicotine administration paradigms, have investigated the influence of nicotine exposure during pregnancy on offspring brain development in rodents, models of NRT during pregnancy, in which nicotine doses yield plasma nicotine levels comparable to those measured in humans have only recently been assessed. In particular, using subcutaneous osmotic mini-pumps to chronically administer high (6 mg/kg/d) or low (2 mg/kg/d) doses of nicotine throughout pregnancy in Sprague-Dawley rats, Hussein and collaborators showed that plasma nicotine levels measured in dams of the high dose group (125–175 ng/ml) were much higher than in moderate to heavy smokers (10–50 ng/ml) [18]. In comparison, plasma nicotine levels in dams from the low dose group were much closer (30–40 ng/ml) to these values for smokers [18]; these values are also similar to plasma levels in abstinent nicotine patch users (approximately 15 ng/ml) [19], [20], making this model particularly relevant to these NRT users.

Here, we have used the model examined by Hussein et al. [18] to explore the consequences of exposure to maternal NRT-like nicotine throughout gestation and breastfeeding on cerebral neuroplasticity in offspring. Our investigation was focused on two forms of neural plasticity that occur throughout life in the dentate gyrus (DG) of the hippocampus: granule cell neurogenesis and long-term potentiation (LTP). DG granule cell neurogenesis is a lifelong neurodevelopmental phenomenon that has been associated with a variety of cerebral functions such as stress regulation [21], [22] and learning and memory [23], [24], and can be persistently affected by early-life environment [25]–[27]. In contrast, LTP is the synaptic mechanism thought to underlie memory formation [28]. We report that rats chronically exposed to NRT-like conditions during both prenatal (*in utero*) and postnatal (breastfeeding) development do not display alterations in DG neurogenesis but show significantly enhanced DG LTP compared to saline-exposed controls.

## Materials and Methods

### Animals

All experiments were approved by McGill University's Animal Care Committee (approval ID: 5367) and followed the policies and guidelines of the Canadian Council on Animal Care. Sprague-Dawley rats were purchased at gestational day 2 (G2) from Charles River (St-Constant, QC, Canada) and kept in separate cages with water and food *ad libitum*. At postnatal day 2 (P2), litters were culled to eliminate litter size-induced variability in pup growth. Offspring weight was recorded regularly until weaning.

### Nicotine and 5-bromo-2′-deoxyuridine administration

At G3, dams were briefly anesthetized with isofluorane and implanted with subcutaneous osmotic mini-pumps (Alzet; model 2006, 6 week duration) filled with sterile saline (n = 9) or a solution of nicotine bitartrate yielding 2 mg free base/kg/d nicotine (n = 7). Nicotine concentration was calculated from actual (as opposed to projected) weight, as described previously [18]. Mini-pumps were primed so as to deliver solutions at a constant rate (0.25 µl/h) from subcutaneous implantation. No dams displayed abnormal recuperation from anesthesia or post-implantation infection. Offspring were thus chronically exposed to nicotine *in utero* between embryonic day 3 (E3) and birth, and then through breastmilk until P21 (weaning). To assess DG cell differentiation and survival, the thymidine analog and DNA synthesis marker 5-bromo-2′-deoxyuridine (BrdU) dissolved in 0.9% NaCl with 0.4 N NaOH was injected in P15 pups (50 mg/kg; i.p.) either once or twice (four hours apart). Animals were then sacrificed by cardiac perfusion either 2 hours or 26 days (P41) later, respectively.

### Plasma nicotine and nicotine metabolism

To determine plasma nicotine and cotinine (a nicotine metabolite) levels in dams during gestation, tail-vein blood was collected at G15 and at 7 days postpartum. Trunk blood was collected from breastfed pups at P7 for the same purpose. Immediately following blood collection, plasma was centrifuged at 3000 *g* for 10 min and frozen at −20°C until analysis by HPLC, as described previously [29]. To compare nicotine metabolism between developing offspring and and adults, livers from P7 and P68 naive rats (n = 5 per age) were extracted, flash frozen, and stored at −20°C until analysis. Liver microsomal membranes were prepared for *in vitro* nicotine metabolism assays as previously described [29], [30] and stored at −80°C in 1.15% KCl. The cytosolic fractions were separated during membrane preparation and used as a source of aldehyde oxidase. Adult liver microsome mixtures contained 0.5 mg/ml protein, 80 µl of nicotine (0–480 µM final concentration), 1 mM NADPH and 50 µl rat liver cytosol in 50 mM Tris-HCl buffer (pH 7.4), and were incubated at 37°C for 20 min in a final volume of 0.5 ml. The reaction was stopped with 100 µl 20% w/v Na_2_CO_3_, giving a final concentration of 4% w/v. After incubation 5-methylcotinine (50 µl of 1.3 µg/ml) was added as the internal standard. For P7 rats the assay volume was scaled down by a factor of four. The samples were prepared and analyzed for nicotine and cotinine by HPLC as described previously; the limits of quantification were 5 ng/ml for nicotine and 12.5 ng/ml for cotinine.

### Plasma corticosterone

To assess baseline stress hormone levels in offspring during postnatal nicotine exposure (n = 6; controls: n = 7), plasma corticosterone was measured by radioimmunoassay from trunk blood in P15 pups using a commercial kit (MP Biomedical) with slight modifications, and allowing for a detection threshold of 0.2 µg/dl, as described previously [31]. In brief, 5 ul of plasma in assay buffer were heated at 65°C for 30 min to denature corticosteroid-binding globulin and then incubated with 125I-Corticosterone and the specific anti-corticosterone antiserum. After overnight incubation at 4°C, the bound corticosterone was separated from the free form by addition of a charcoal (6.25 g/L)-dextran (0.625 g/L) solution. 15 min after addition of the charcoal solution, tubes were centrifuged at 4°C for 30 min (3000 rpm). The supernatant containing the bound fraction was collected into glass tubes and counted in a gamma counter.

### Cardiac perfusions

At P15 (juveniles) or P41 (adolescents), offspring were perfusion-fixed for histological processing of their brains. Animals were deeply anesthetized with a cocktail of ketamine (50 mg/kg), xylazine (5 mg/kg) and acepromazine (1 mg/kg), and intracardially perfused with ice-cold phosphate-buffered saline (PBS; 50 mM) followed by 4% formaldehyde in 0.1 M phosphate buffer. Brains were then rapidly removed, postfixed overnight at 4°C in fixative and transferred to a 30% sucrose solution until equilibrium was reached. Brains were sliced using a cryostat into serial 40 µm-thick coronal sections that were placed in a cryoprotectant solution (glycerol∶glycol∶PBS, 3∶3∶4) and stored at −20°C until processed for free-floating immunohistochemistry, as described below.

### Ki-67 immunocytochemistry

All immunohistochemistry incubations were performed at room temperature unless otherwise specified. PBS washes preceded all steps aside from addition of primary antibodies. Cell proliferation in the DG was quantified as numbers of cells immuonoreactive (IR) for Ki-67, a protein expressed in the nucleus of proliferating cells. Sections were mounted on slides in PBS containing 0.2% Tx-100 (PBS-T) and incubated in 10 mM sodium citrate buffer (pH 6, 95°C) for 15 min, then incubated in 3% H_2_O_2_ in PBS for 10 minutes and in 2% normal horse serum diluted in PBS-T for 30 min. Sections were then incubated overnight at 4°C with monoclonal mouse anti-Ki-67 antibody (1∶2000; Pharmingen) in the same solution, followed by a 1 h incubation in biotinylated horse anti-mouse antibody (1∶200; Vector Laboratories) in this solution, and the avidin-biotin complex procedure (ABC Kit, Vectastain Elite; Vector Laboratories) for 30 min. Labeling was revealed with a DAB kit (Vector Laboratories) for 5 min. Sections were then dehydrated and coverslipped using Permount (Fisher Scientific).

### BrdU immunocytochemistry

Numbers of BrdU-IR cells at P41 (more than 3 weeks after BrdU administration at P15) were used to asses DG cell survival. Sections were incubated for 2 h in PBS-T, 10 min in 0.9% H_2_O_2_ in PBS, and 30 min at 37°C in 2N HCl in PBS to denature DNA. Sections were then incubated for 30 min in PBS-T containing 2% normal goat serum and overnight at 4°C with monoclonal rat anti-BrdU (1∶1000; Serotec) in the same solution. Sections were then incubated for 1 h in this solution with biotinylated goat anti-rat antibody (1∶200; Vector Laboratories), followed (as above, unless otherwise specified) by the ABC and DAB procedures (2 min), dehydration, mounting on slides, and coverslipping.

### BrdU/NeuN double-labeling immunocytochemistry

To determine the proportion of newborn cells that differentiated into neurons, a subset of coronal brain sections across the dorsal DG of BrdU-injected offspring (P41) was double-labeled for BrdU and NeuN (a neuron-specific nuclear marker). Sections were incubated in rat anti-BrdU (1∶1000) and mouse anti-NeuN (1∶200; Chemicon) antibodies, after consecutive incubations in PBS-T (2 h), 2 N HCl in PBS (30 min), and PBS-T containing 2% normal goat serum (1 h). This was followed by a 1.5 h incubation in fluorescent DyLight 594-labeled goat anti-rat (1∶1500; Jackson) and DyLight 488-labeled goat anti-mouse (1∶500; Jackson) antibodies diluted in 2% NGS in PBS-T. Sections were mounted on glass slides and coverslipped with ProLong Gold antifade reagent with DAPI (Invitrogen).

### Cell quantifications

The section-sampling fraction for cell proliferation and survival experiments was 1/8. For each animal, Ki-67-IR and BrdU-IR cells were counted in the granule cell layer and adjacent subgranular zone across the entire dorsal DG (corresponding to Bregma -2.4 mm to -4.8 mm [32]), using an Olympus BX51 light microscope.

BrdU/NeuN imaging was performed as described previously [33], using a Zeiss LSM510 Meta confocal microscope with an Axiovert 200 M stand and motorized stage (Carl Zeiss Canada), with 488 nm and 543 nm wavelength lasers, at the Cell Imaging and Analysis Network (CIAN) facilities at McGill University, Montreal. For each animal, at least ten randomly-selected BrdU-IR cells from multiple DG sections were analyzed (n = 5/group), and data expressed as the percentage of BrdU-IR cells that were also NeuN-IR. Images were not altered except for overall brightness and contrast. All cell quantifications were conducted by an experimenter blinded to experimental group identity.

### Basal glutamatergic transmission in the DG

To evaluate the consequences of chronic developmental NRT-like nicotine exposure from breastmilk on basal glutamatergic transmission in the hippocampal DG of juvenile pups, we compared the relationship between presynaptic fiber volley amplitude, which represents the number of presynaptic fibers firing action potentials, and the slope of field excitatory post-synaptic potentials (fEPSPs) in the DG. P15 offspring (saline-exposed: n = 6; nicotine-exposed: n = 5) were anesthetized as above and sacrificed to prepare 400 µm-thick coronal brain slices, as described previously [34], [35]. fEPSPs were evoked by stimulating the medial perforant pathway using a constant current pulse and recorded from the terminal region of the medial perforant pathway. fEPSPs were amplified using a Multiclamp 700B amplifier (Axon), digitally stored, and analyzed off-line. Slices displaying unstable baseline recording (±10%) were discarded. fEPSP slopes recorded from saline- and nicotine-exposed rats were normalized by fiber volley amplitude and compared.

### Long-term potentiation in the DG

In addition to granule cell neurogenesis, LTP is another major form of functional plasticity in the DG. We therefore sought to determine if this phenomenon is affected in juvenile rats exposed to nicotine through breastfeeding. LTP of fEPSPs in the hippocampal slice preparations described above were induced by theta-burst stimulation (TBS), as a ten-burst train of four pulses (100 Hz) with 200 ms inter-train intervals [36]. Bicuculline methiodide (10 µM) was included in extracellular artificial cerebrospinal fluid (aCSF) to block GABA_A_ receptor-mediated inhibitory post-synaptic currents (IPSCs). Changes in fEPSPs after TBS were monitored for at least 60 min.

### Statistics

Unless specified otherwise, results in each experiment were generated from one male offspring per litter and from 5–7 litters per group and are expressed as means ± SEM. Electrophysiological data (n = number of recordings) were obtained from 3–4 male offspring from different litters. Pairwise comparisons were performed using two-tailed t-tests with a threshold for significance of p<0.05. Relationship between fEPSP slope and fiber volley amplitude was examined by a regression analysis.

## Results

### Litter size and pup growth

**Figure 1:**
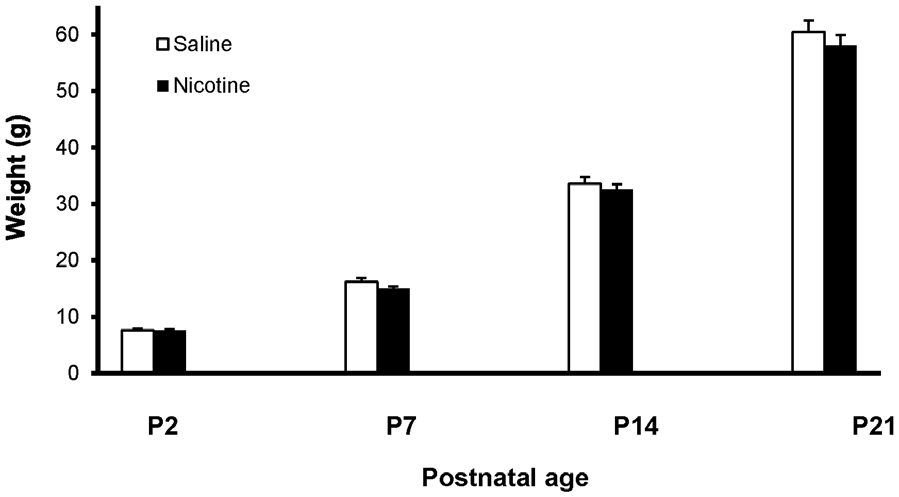
Offspring Weight. Between postnatal day 2 (P2) and weaning (P21), the average weight of male pups exposed to maternal nicotine did not differ significantly from that of saline-exposed controls (ps = 0.16–0.94).

Litters chronically exposed to nicotine or saline revealed no significant difference in the average number of pups (saline: 13.2±1.0 vs nicotine: 12.6±0.8; p = 0.63), or in the number of males (saline: 6.2±1.0 vs nicotine: 7.3±1.0; p = 0.44) or females (saline: 7.0±0.9 vs nicotine: 5.3±1.0; p = 0.22) per litter. Similarly, average pup weight did not display any significant variation between saline- and nicotine-exposed pups at any age examined between P2 and weaning (ps = 0.16–0.94) (**Fig. 1**). These results are consistent with data previously obtained with the same model by Hussein and colleagues [18].

### Plasma nicotine and cotinine measurements

**Figure 2:**
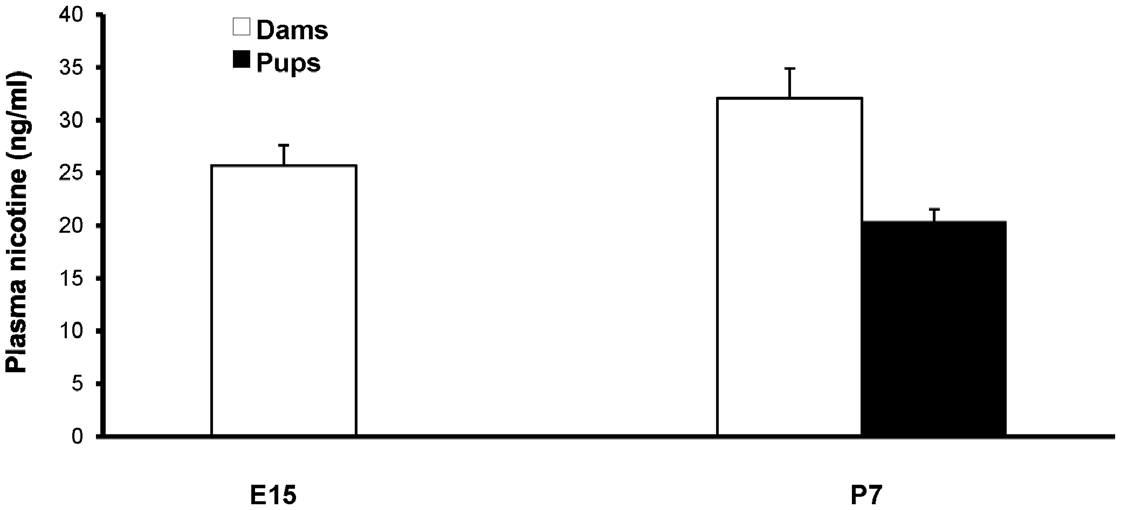
Plasma nicotine levels in nicotine-exposed dams and their breastfed pups. Dam nicotine levels increased non-significantly between pregnancy and post-parturition (p = 0.14). E15: embryonic day 15; P7: postnatal day 7 days post-parturition.

As illustrated in **Fig. 2**, plasma nicotine levels in dams measured early in the third week of embryonic development (E15; n = 7) averaged 25.7±1.9 ng/ml. This value increased slightly (but non-significantly; p = 0.14) to 32.1±2.8 ng/ml at 7 days postpartum, during breastfeeding. In P7 male pups, plasma nicotine levels averaged 20.43±1.1 ng/ml (n = 7 litters, average of 2 pups/litter). The levels of cotinine were relatively high in dams, at 232.1±25.4 ng/ml at G15 and 163.9±9.6 ng/ml at 7 days postpartum, with the postpartum value being significantly lower (p = 0.02; not illustrated) than during pregnancy. Despite easily detectable plasma nicotine levels at P7, offspring cotinine was undetectable in these plasma samples.

### Nicotine metabolism

In order to investigate the differences in plasma levels of cotinine observed in the P7 pups relative to the adults, the *in vitro* rates of nicotine-to-cotinine metabolism were assessed. Liver microsomes from adult rats metabolized nicotine to cotinine with an apparent affinity constant (Km) of 169±21 µM, similar to the apparent Km in P7 rat pups of 172±24 µM (n = 5 different rats for each age group, assayed twice). However, when the velocity of this reaction was compared, the adult liver microsomes metabolized nicotine with a substantially faster velocity; nicotine concentrations near to the apparent Km (120 µM) resulted in velocities of 0.181±0.025, compared to P7 rat pup microsomes of 0.024±0.004 nmol/min/mg protein (p<0.001). The apparent Vmax was also substantially higher in adult vs P7 rats (0.426±0.015 vs 0.057±0.010 nmol/min/mg protein, p<0.001).

### Plasma corticosterone in breastfed pups

**Figure 3:**
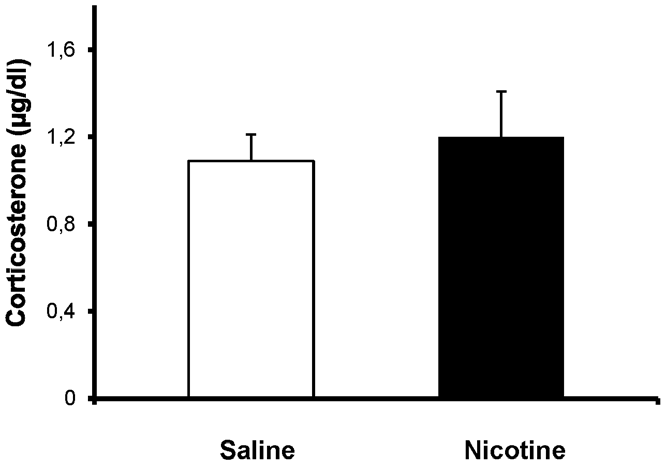
Plasma corticosterone in pups. Basal plasma corticosterone measured in juvenile pups exposed through breastfeeding to nicotine or saline (controls) from early embryogenesis. Corticosterone levels did not differ between groups (p = 0.67).

As shown in **Fig. 3**, chronic nicotine exposure did not affect plasma corticosterone levels in P15 male pups; 1.19±0.21 µg/ml (n = 6 litters; 2pups/litter) compared to 1.08±0.12 µg/ml (n = 7 litters; 2 pups/litter) in controls (p = 0.67). These results suggest that chronic NRT-like exposure during prenatal and postnatal development does not alter basal stress hormone levels in pups.

### DG cell proliferation and neurogenesis

**Figure 4:**
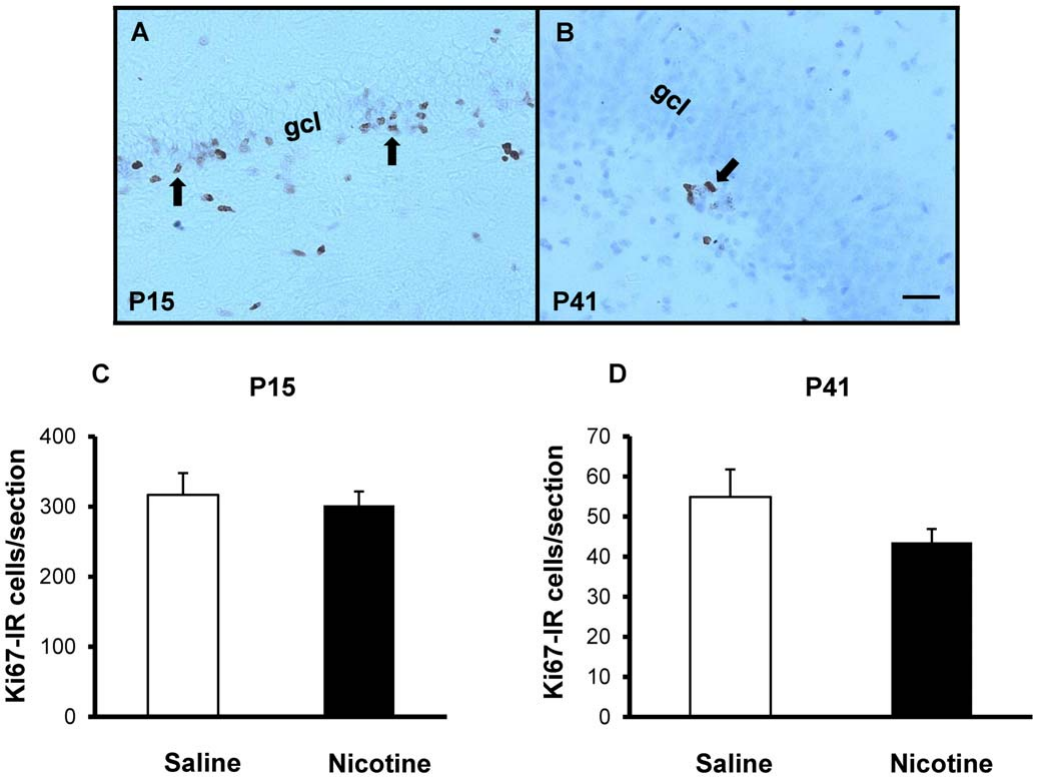
Cell proliferation. DG proliferation assessed from Ki67-immunostained brain sections from juvenile (P15) and adolescent (P41) male offspring exposed to saline (controls) or nicotine throughout prenatal and postnatal development. Representative micrographs of Ki67-IR cells (arrows) in the subgranular zone or adjacent granule cell layer (gcl) in control P15 (**A**) and P41 (**B**) offspring. No significant difference in numbers of Ki67-IR cells was found in the dorsal hippocampus of P15 (**C**; p = 0.70) or P41 (**D**; p = 0.17) offspring. Scale bar = 25 µm.

**Figure 5:**
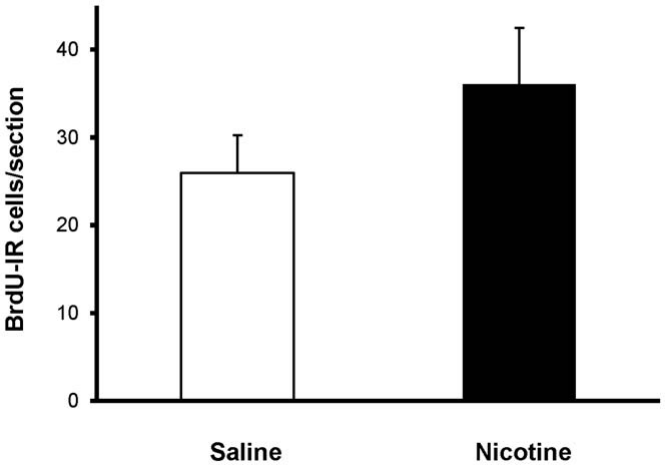
Cell survival. Developmental nicotine exposure did not affect survival of DG cells (p = 0.22). Survival of newborn cells was estimated from BrdU-immunostained brain sections in adolescent (P41) offspring exposed to saline (controls) or nicotine from early embryogenesis until weaning and injected with BrdU at P15.

**Figure 6:**
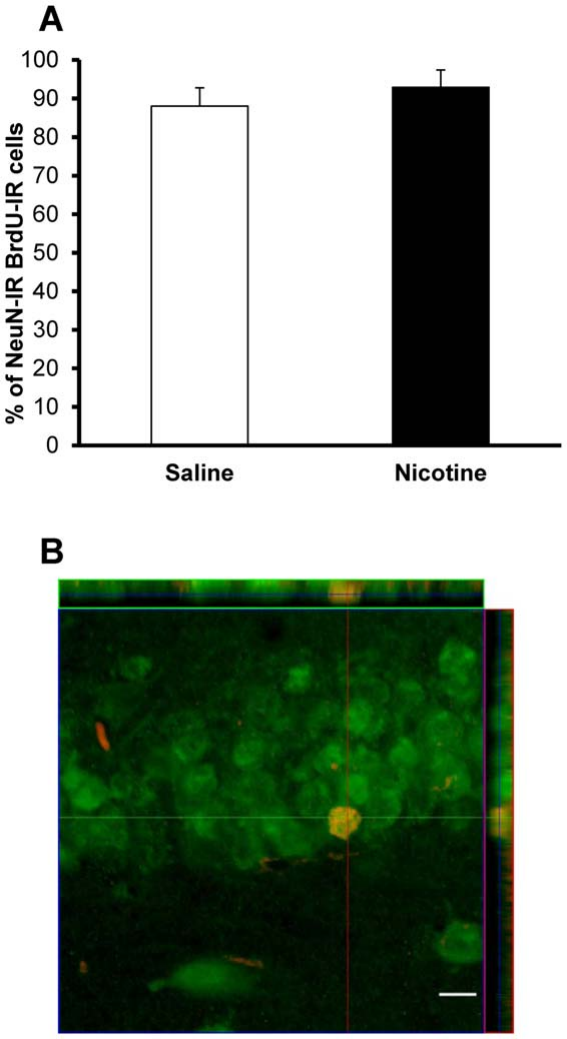
Neuronal differentiation. (**A**) Proportion of BrdU-IR cells differentiating into neurons in adolescent (P41) offspring exposed to saline (controls) or nicotine from early embryogenesis until weaning and injected with BrdU at P15 did not differ between groups (p = 0.48). (**B**) Orthogonal confocal image of a BrdU/NeuN-labeled DG cell (red: BrdU; green: NeuN). Scale bar = 10 µm.

Numbers of Ki-67-IR cells in the dorsal DG did not differ significantly between saline- and nicotine-exposed offspring at either P15 (316.94±30.82 vs 301.97±19.70 cells, respectively; p = 0.70) or P41 (54.88±6.87 vs 43.55±3.38 cells, respectively; p = 0.17) (**Fig. 4**). Cell survival, as assessed by the number of BrdU-IR cells present in the DG of P41 rats that were administered BrdU at P15 was similar in nicotine-exposed and control groups (25.98±4.26 vs 36.08±6.38 cells, respectively; p = 0.22) (**Fig. 5**). In addition, the BrdU/NeuN confocal analysis revealed that, nearly four weeks after BrdU administration, roughly the same proportion of BrdU-IR cells had adopted a neuronal phenotype (control group: 88±4.9%; nicotine-exposed group: 92±4.5%; p = 0.48; **Fig. 6**). Taken together, these data suggest that chronic NRT-like nicotine exposure during embryonic development and breastfeeding does not alter DG cell proliferation, differentiation or survival in juvenile or adolescent offspring.

### Glutamatergic synaptic transmission and LTP in the DG

**Figure 7:**
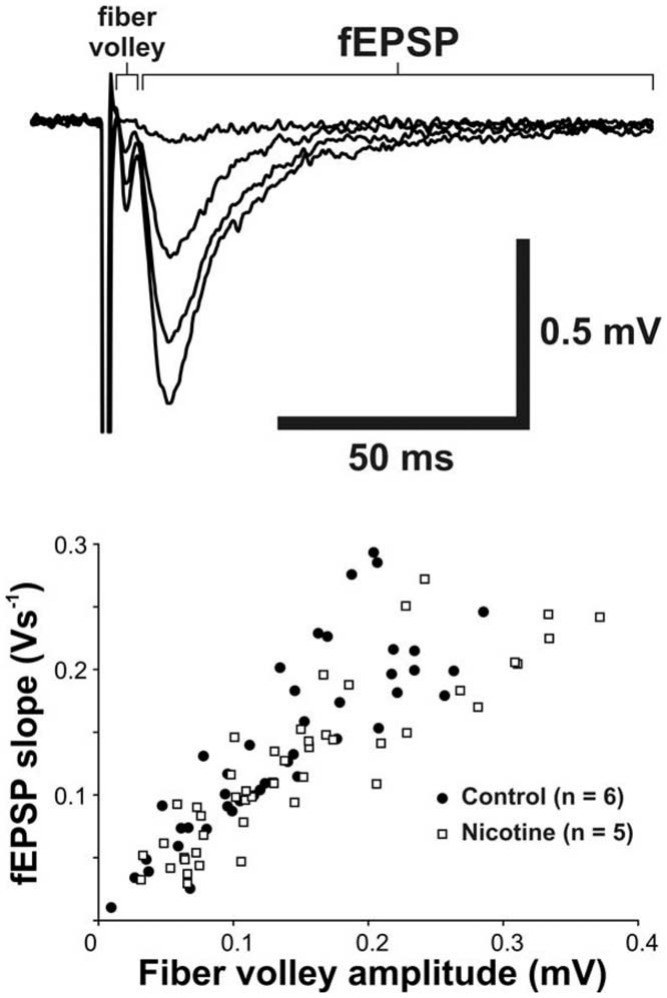
Basal synaptic transmission. Basal excitatory synaptic transmission was not affected by nicotine exposure. Upper panel: averaged traces of evoked field excitatory postsynaptic potential (fEPSP) recorded from the DG of P15 rat pups. The small depolarization ahead of fEPSP is the fiber volley, which represents the amount of activated presynaptic fibers. Basal excitatory synaptic transmission is therefore related to the ratio of fiber volley vs fEPSP at different stimulating currents (see the four averaged traces). We compared this ratio between control and nicotine-treated pups and found no differences between these two groups (lower panel: comparing slope of regression lines fitting fEPSPs and fiber volley amplitudes between two groups, p = 0.21).

**Figure 8:**
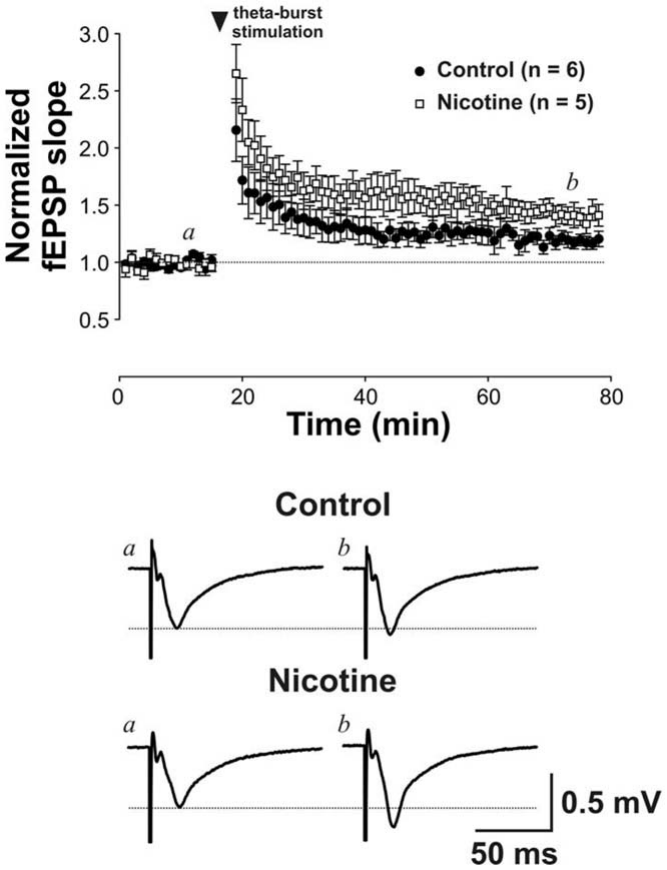
Long-term potentiation. Long-term potentiation was enhanced by nicotine exposure. Upper panel: Scattered plots of fEPSP against time revealed changes in fEPSP after long-term potentiation (LTP) induction by theta-burst stimulation. Note that LTP triggered in pups exposed to nicotine was stronger than that in control pups. Lower panel: Average traces of fEPSP obtained at time *a* (before LTP induction) and *b* (60 min after LTP induction) in representative recordings obtained from saline- and nicotine-exposed pups (% potentiation at 60 minutes after TBS: p = 0.037).

The regression lines fitting fEPSPs and fiber volley amplitudes for saline- and nicotine-exposed animals were indistinguishable (F(2,88) = 1.62, p = 0.21; **Fig. 7**). However, LTP induced by TBS was robustly increased in nicotine-exposed animals, suggesting that nicotine ingested from breastmilk increases synaptic plasticity in the immature DG (potentiation at 60 minutes after TBS: control group: 18.2±5.5%; nicotine-exposed group: 40.8±8.7%; p = 0.037; **Fig. 8**).

## Discussion

The model of NRT during pregnancy and breastfeeding used in this study was chosen because it (1) mimics nicotine delivery through transdermal patches, the most commonly employed NRT, (2) results in maternal plasma nicotine levels similar to those measured in humans [18], and (3) exposes offspring to nicotine throughout prenatal and postnatal development, a period encompassing DG development [37], [38]. Postnatal nicotine exposure in pups was assumed on the basis of previous reports showing that nicotine diffuses into breast milk [39] and upregulates brain nicotinic acetylcholine receptors (nAChRs) in breastfed pups [40]. This maternal transfer of nicotine to breastfed pups was confirmed here by showing that plasma nicotine levels in juvenile offspring are comparable to those found in their mothers, with both in the range of those measured in adult humans using NRT [20]. Based on the results of our enzymatic analyses, the relatively high levels of circulating nicotine we observed in offspring were likely due to a combination of absorbance of low levels from breast milk combined with a considerably reduced rate of metabolism of nicotine to cotinine during the postnatal period, as indicated by both a lack of detection of plasma cotinine in the pups and decreased *in vitro* enzyme activity. These data in our rat model are consistent with the substantially longer nicotine half-life in human newborns (3–4 times longer than adults) [41], and provide the first evidence that this is likely due to decreased hepatic nicotine-to-cotinine metabolism.

In agreement with previous reports in rodents [18] and humans [17], we found no effect of developmental exposure to NRT-like nicotine levels on pregnancy outcome or postnatal weight. Several nAChR subunits are strongly expressed in the hippocampus and other brain areas as early as mid-embryogenesis [42]–[44]. These subunits have been shown to form functional receptors in different regions of the immature central nervous system [42], [45], suggesting that nicotine exposure interferes with neuronal development and affects brain and behavior in the long-term. This hypothesis has given rise to an extensive literature on chronic nicotine exposure and its consequences on brain development. In particular, many rodent studies have been published using a variety of nicotine administration paradigms, varying with respect to route, dose, duration and timing (e.g. [46]–[52]). Although these studies have predominately shown that certain aspects of brain development are altered by developmental nicotine exposure, inconsistent methodology has made it difficult to fully appreciate their relevance to humans, particularly in relation to NRT. Thus, the aim of this study was to use and further validate in pups an NRT-like administration paradigm to examine DG neurogenesis and LTP, two highly neuroplastic phenomena known to be sensitive to the environment.

Interestingly, we found that NRT-like nicotine exposure had no significant influence on DG neurogenesis, from cell proliferation to neuronal fate and survival. With respect to proliferation, these findings are in line with previous studies showing that DG proliferation is unaltered by short-term nicotine administration [53]. The findings regarding survival were unexpected given previous reports showing that nicotine reduces survival of hippocampal progenitors *in vitro* [54], and that adult rats self-administrating nicotine for 42 days display decreased neurogenesis [55]. To our knowledge, the current study is the first to have examined DG neurogenesis in immature rats exposed to nicotine. To model the influence of NRT-like nicotine during breastfeeding, rats were exposed to nicotine until weaning (P21). Although it remains possible that continued nicotine exposure until sacrifice (P42) could have altered newborn DG cell phenotype and survival, as documented in adults by Abrous and colleagues [55], the present data suggest that nicotinic regulation of cell proliferation and neurogenesis in the juvenile/adolescent DG differs from that of adults. This may be related to differential expression of α7 nAChRs, which regulate the maturation, integration and survival of adult-born DG neurons [56]. It remains to be determined whether DG neurogenesis in adults is influenced by early NRT-like nicotine exposure; however, the lack of neurogenic effects seen in the present study suggests that later stages may be unaffected as well.

Prior to this study, NRT-like nicotine exposure during gestation and early development had not been assessed for its consequences on synaptic plasticity. In the absence of changes in DG neurogenesis or fEPSPs in offspring, we found that developmental NRT-like exposure significantly increased LTP in the DG of juvenile pups. These results suggest that NRT-like nicotine exposure *in utero* and from breastmilk enhances synaptic plasticity in the immature DG. Nicotine administration increases expression of cyclic AMP response element binding protein (CREB), a transcription factor associated with plasticity, in hippocampal neurons [57], suggesting that changes in CREB activation or downstream transcription may underlie the LTP changes seen in the current study. It would be interesting for future investigations aimed at exploring the molecular mechanisms underlying the enhanced LTP measured in this study to examine the expression of CREB and other plasticity-related markers, such as Zif268 [58], in the hippocampus of NRT-like exposed offspring. It is well known that LTP in the DG is highly sensitive to exogenous stimuli [59], [60], and it has previously been shown to be enhanced *ex vivo* in the hippocampi of adult rats chronically exposed to nicotine [61]. The mechanisms underlying nicotinic regulation of LTP induction in the DG thus seem conserved throughout life. It is unknown at present whether the increased LTP observed in juveniles persists later in life, but it can be speculated that this significant alteration in synaptic plasticity during a critical period of postnatal development causes lasting changes to hippocampal circuitry. Supporting this notion, hippocampal LTP during development is known to drive the formation of glutamatergic synapses [62]. It is possible that by enhancing synaptogenesis, early-life NRT exposure would affect hippocampus-dependent cognitive functions, such as learning and memory. Although increases in hippocampal LTP are traditionally associated with increased memory performance [63], its occurrence during development may be functionally maladaptive [64]. In this context, it is interesting to note that learning and memory deficits [5], [6] have been associated with maternal smoking. Given that these effects may be partly attributable to nicotine exposure during early development, it has been suggested that NRT is not an ideal therapy for pregnant smokers, as it is not sufficiently efficacious, harmless or superior to non-pharmacological smoking cessation therapies [65], [66]. Although the NRT model used in the present study does not suggest harmful consequences of early-life NRT exposure on pregnancy outcome or postnatal growth, it does reveal changes affecting connectivity in the growing brain. Future behavioral studies using this model will need to explore whether such changes are long-lasting, whether they affect other molecular and physiological aspects of hippocampal plasticity, and whether they are associated with altered cognition or affective behavior.
